# Flavopereirine Suppresses the Growth of Colorectal Cancer Cells through P53 Signaling Dependence

**DOI:** 10.3390/cancers11071034

**Published:** 2019-07-22

**Authors:** Jhy-Ming Li, Yun-Ching Huang, Yi-Hung Kuo, Chih-Chung Cheng, Feng-Che Kuan, Shun-Fu Chang, Ying-Ray Lee, Chih-Chien Chin, Chung-Sheng Shi

**Affiliations:** 1 Division of Colon and Rectal Surgery, Department of Surgery, Chang Gung Memorial Hospital, Chiayi 61363, Taiwan; 2 Graduate Institute of Clinical Medical Sciences, College of Medicine, Chang Gung University, Taoyuan 33302, Taiwan; 3 Division of Urology, Department of Surgery, Chang Gung Memorial Hospital, Chiayi 61363, Taiwan; 4 Department of Hematology and Oncology, Department of Medicine, Chang Gung Memorial Hospital, Chiayi 61363, Taiwan; 5 Department of Medical Research and Development, Chang Gung Memorial Hospital Chiayi Branch, Chiayi 61363, Taiwan; 6 Department of Medical Research, Ditmanson Medical Foundation Chia-Yi Christian Hospital, Chiayi 60002, Taiwan

**Keywords:** colorectal cancer, Flavopereirine, P53

## Abstract

Colorectal cancer (CRC) is a significant cause of morbidity and mortality worldwide. The outcome of CRC patients remains poor. Thus, a new strategy for CRC treatment is urgently needed. Flavopereirine is a β-carboline alkaloid extracted from *Geissospermum vellosii*, which can reduce the viability of various cancer cells through an unknown mode of action. The aim of the present study was to investigate the functional mechanism and therapeutic potential of flavopereirine on CRC cells in vitro and in vivo. Our data showed that flavopereirine significantly lowered cellular viability, caused intrinsic and extrinsic apoptosis, and induced G2/M-phase cell cycle arrest in CRC cells. Flavopereirine downregulated Janus kinases-signal transducers and activators of transcription (JAKs-STATs) and cellular myelocytomatosis (c-Myc) signaling in CRC cells. In contrast, the enforced expressions of constitutive active STAT3 and c-Myc could not restore flavopereirine-induced viability reduction. Moreover, flavopereirine enhanced P53 expression and phosphorylation in CRC cells. CRC cells with P53 knockout or loss-of-function mutation significantly diminished flavopereirine-mediated viability reduction, indicating that P53 activity plays a major role in flavopereirine-mediated CRC cell growth suppression. Flavopereirine also significantly repressed CRC cell xenograft growth in vivo by upregulating P53 and P21 and inducing apoptosis. In conclusion, flavopereirine-mediated growth suppression in CRC cells depended on the P53-P21, but not the JAKs-STATs-c-Myc signaling pathway. The present study suggests that flavopereirine may be efficacious in the clinical treatment of CRC harboring functional P53 signaling.

## 1. Introduction

High-grade colorectal cancer (CRC) is a major cause of morbidity and mortality worldwide [1]. Though advances have been made in current therapeutic approaches, their benefits are limited in clinical practice [2,3]. Thus, searching for a new strategy for CRC treatment is urgently needed. The pathogenetic mechanisms underlying CRC development and progression are complex and heterogeneous [4,5]. Risk factors in CRC development include the influences of early-stage adenomatous polyposis coli (*APC*) mutations [6] and late-stage tumor suppressor *p53* and *K-ras* mutations on malignant epithelial-mesenchymal phenotype transformation [7].

Various signaling pathways such as Janus kinases-signal transducers and activators of transcription (JAKs-STATs) participate in CRC progression [8]. When cytokines bind to their receptors, JAKs and STATs are recruited and phosphorylated, resulting in the nuclear translocation of dimerized STATs and the expression of downstream mediators [9]. The four mammalian JAK family members are JAK1, JAK2, JAK3, and Tyrosine Kinase 2. They selectively phosphorylate and activate STATs [10]. Moreover, certain growth factor receptors also induce STATs by promoting intrinsic tyrosine-kinase activation including the epidermal growth factor [11], G-protein-coupled, and Toll-like [9] receptors. STAT1, STAT2, STAT3, STAT4, STAT5a, STAT5b, and STAT6 are also identified in mammalian cells [12]. Of these, STAT3 has been studied the most. It increases tumor cell proliferation, differentiation, metastasis, and angiogenesis by stimulating tumor-promoting inflammation [13]. STAT3 activation is also involved in CRC tumorigenesis [14]. It regulates B-cell lymphoma 2 (Bcl-2), Myeloid cell leukemia-1 (Mcl-1), and cellular myelocytomatosis (c-Myc) expression to maintain cell survival and proliferation [15,16]. Specific siRNAs or small molecular inhibitor of STAT3 can sensitize CRC cells to chemoradiotherapy in vitro and in vivo [17].

P53 plays an important role in the cellular stress response pathways for responding to DNA damage and repair, apoptosis, and senescence [18,19]. It transcriptionally activates downstream genes for inducing cell cycle arrest and apoptosis [20,21]. P53 is upregulated and its phosphorylation increases in response to DNA damage. This increased P53 activity regulates P21 expression, induces cell cycle arrest, interferes with DNA replication, and repairs DNA breaks [22]. Several P53-dependent target genes, including *p21* and *bax*, are identified by sequence-specific transcriptional analysis [23]. P53 can bind directly to Bcl-2 and B-cell lymphoma-extra large (Bcl-XL), which prevents their mitochondrial translocation via losing out mitochondrial membrane integrity and releases cytochrome *c* for inducing cellular apoptosis [24]. Moreover, *p53* mutation is frequently associated with poor clinical outcomes in various human malignancies [25]. In CRC, it is observed in ~40% to 50% of all CRC cases [19] and causes tumor progression and treatment resistance. Thus, targeting and restoring P53 activity are promising tumor therapy enhancement strategies [26,27,28].

Flavopereirine (3-ethyl-12H-indolo[2,3-a]-quinolizinium perchlorate) is a natural β-carboline alkaloid extracted from *Geissospermum vellosii* (Pao pereira) [29]. A previous study shows that flavopereirine selectively inhibits DNA synthesis in cancer cells [30]. The anticancer agent PB-100 which contains flavopereirine and dihydroflavopereirine, selectively inhibits the proliferation of CRC cells but not their normal counterparts [31]. Flavopereirine lowers the viability of drug-resistant glioblastoma cells and suppresses their IL-6-activated proliferation [32,33]. However, the mechanisms and in vivo therapeutic efficacy of flavopereirine in CRC cells are unknown. In the present study, the mode of action and therapeutic potential of flavopereirine in the suppression CRC cell growth via the P53-P21 and JAKs-STATs-cMyc signal pathways were evaluated in vitro and in vivo.

## 2. Results

### 2.1. Flavopereirine Reduces the Viability of Certain CRC Cell Lines

**Figure 1:**
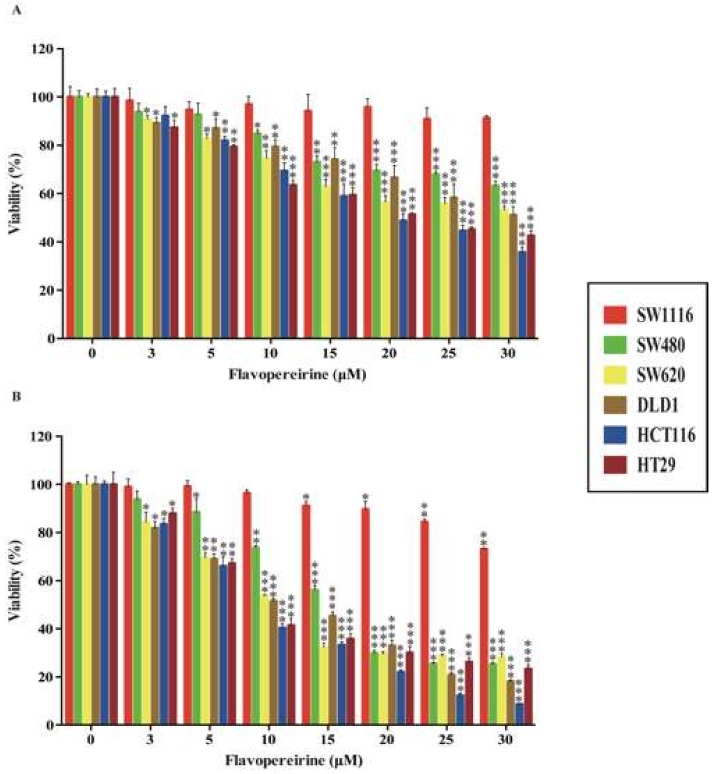
Flavopereirine lowered the viability of various CRC cell lines. CRC cells were treated with various concentrations of flavopereirine for 24 h and 48 h and their viability was assayed by CCK8. Values were expressed as cell viability [%]. Each value represented the mean ± SD of quadruplicate assays. * *p* < 0.05; ** *p* < 0.01, *** *p* < 0.001 compared with the control. Similar results were obtained for at least three independent experiments.

PB100 suppresses the proliferation of CRC cells, but not normal cells [31]. However, the molecular mechanism of flavopereirine in CRC cell growth remains unknown. Here, we investigated the effects of flavopereirine on the viability of various malignant stages of the CRC cell lines SW1116 (Duke’s stage A), SW480 (Duke’s stage B), DLD1, SW620 (Duke’s stage C), HCT116 (Duke’s stage D), and HT29 (no informative Duke’s stage). Figure 1A shows that flavopereirine significantly lowered the viability of SW480, SW620, DLD1, HCT116, and HT29 cells (IC_50_ 58.61, 30.99, 32.37, 19.66, and 21.06 μM, respectively) after 24 h, but had no significant influence on early-stage SW1116 cells. Figure 1B shows that after 48 h treatment with flavopereirine, SW480, SW620, DLD1, HCT116, and HT29 cell viability were significantly reduced (IC_50_ 15.33, 10.52, 10.76, 8.15, and 9.58 μM, respectively). In contrast, it only slightly affected that of SW1116 cells. Moreover, beside HCT116 cells, SW480, SW620, DLD1 and HT29 were relatively resistant to oxaliplatin treatment in the viability assay (Supplementary Figure S1A,B). However, they were very sensitivity to flavopereirine. Thus, flavopereirine may potentially suppress the relatively more malignant and drug-resistant CRC cell lines.

### 2.2. Flavopereirine Promotes Intrinsic and Extrinsic Apoptosis in CRC Cells

**Figure 2:**
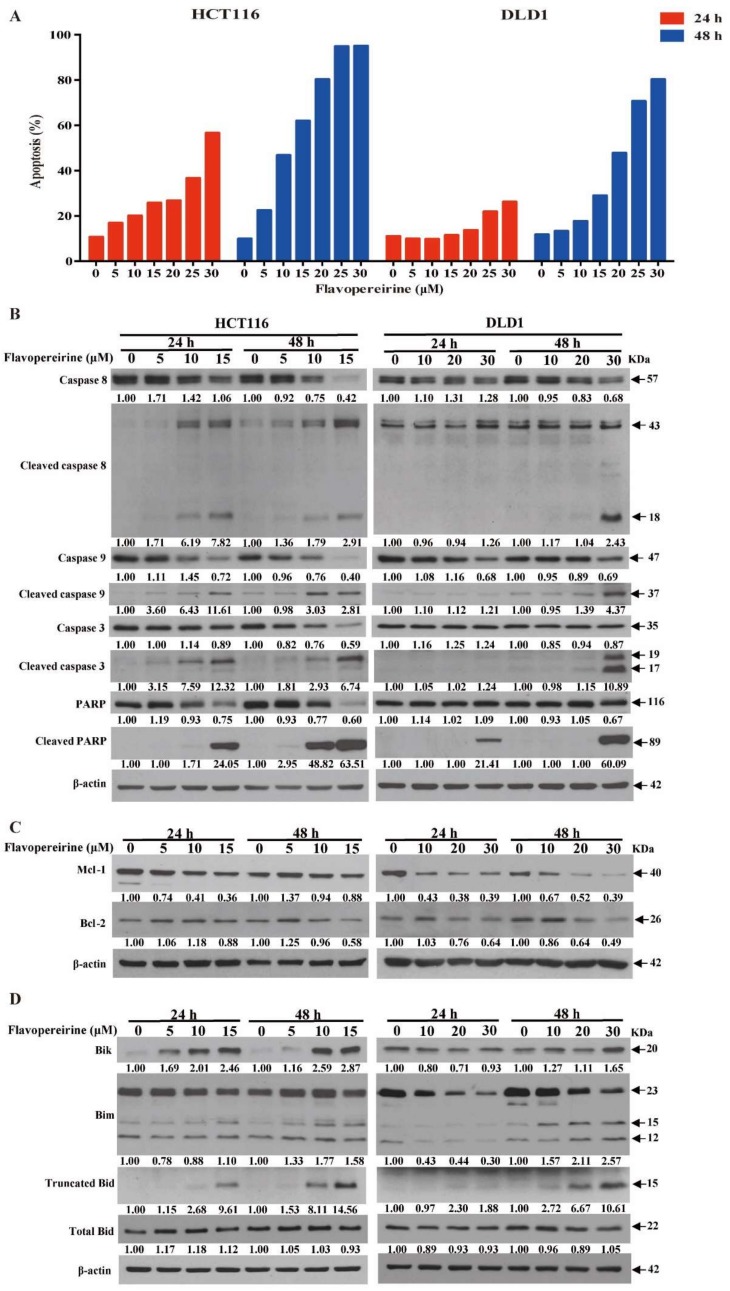
Flavopereirine induced both extrinsic and intrinsic apoptosis in CRC cells. (**A**) Flavopereirine-induced apoptosis in HCT116 and DLD1 cells was assayed and quantified after 24 h and 48 h treatment. (**B**) Flavopereirine promoted the cleavage of caspase-8, -9, and -3 and PARP. (**C**,**D**) Flavopereirine downregulated the pro-survival Bcl-2 family proteins and upregulated the pro-apoptotic proteins Mcl-1, Bcl-2, Bik, Bim, and Truncated Bid. Numbers under the plots indicate the fold change of protein level relative to their untreated control. Similar results were obtained in at least three independent experiments.

As flavopereirine reduced the viability of certain malignant CRC cells, we examined the apoptotic effect of flavopereirine on the most sensitive cell lines. Figure 2A shows that flavopereirine markedly increased both early and late apoptosis in HCT116 and DLD1 after 24 h and 48 h exposure. HCT116 cells were more sensitive to flavopereirine than DLD1 cells in terms of apoptosis induction. Nevertheless, the mechanism involved has not yet been elucidated.

As flavopereirine induced apoptosis in CRC cells, we attempted to determine its underlying mode of action. Figure 2B shows that flavopereirine enhanced the cleavage of caspase-3, -8, and -9, and poly (ADP-ribose) polymerase (PARP) in HCT116 and DLD1 cells after 24 h and 48 h exposure in a dose-dependent manner. Moreover, the pro-survival Bcl-2 family proteins Mcl-1 and Bcl-2 were downregulated after 24 h and 48 h flavopereirine treatment (Figure 2C). In contrast, the expression of Bcl-2 interacting killer (Bik) (the alternate splicing of Bcl-2-like protein 11 (Bim)) and the truncation of BH3 interacting-domain death agonist (Bid) (pro-apoptotic) were upregulated after 24 h and 48 h flavopereirine treatment (Figure 2D). Figure 2A–D demonstrate that flavopereirine induced CRC cell apoptosis via intrinsic and extrinsic pathways in a dose-dependent manner.

### 2.3. Flavopereirine Stimulates G2/M-Phase Cell Cycle Arrest in CRC Cells

**Figure 3:**
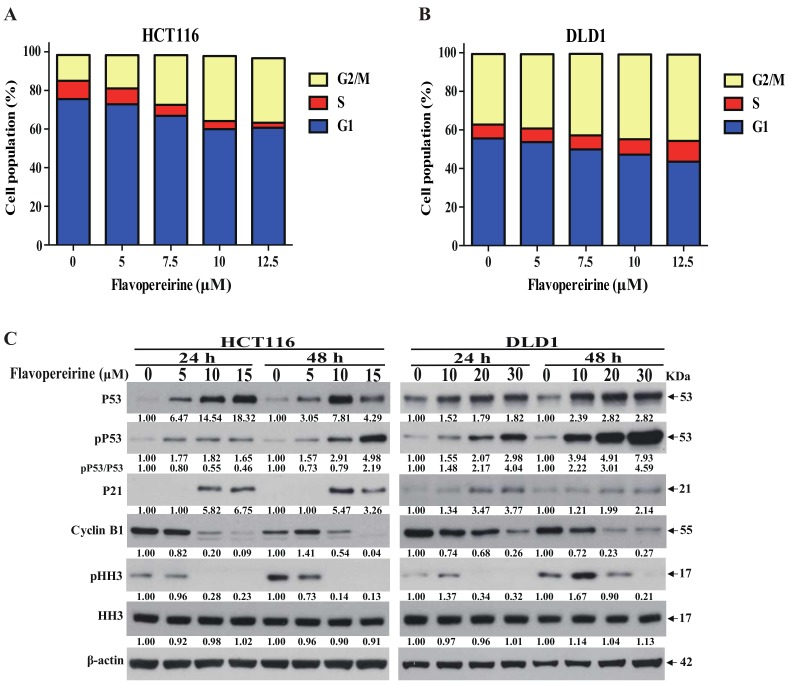
Flavopereirine induced G2/M-phase cell cycle arrest. (**A**) The cell cycle distributions of HCT116 and DLD1 were assayed and quantified after flavopereirine treatment for 24 h. Values were expressed as cell population [%]. (**B**) Flavopereirine upregulated P53 and P21 expression and phosphorylation, downregulated cyclin B1, and diminished the phosphorylation of histone H3 after 24 h and 48 h treatment. Numbers under the plots indicate the fold change of protein level relative to their untreated control. Similar results were obtained for at least three independent experiments.

Flavopereirine significantly constrained CRC cell viability. Thus, we assessed whether flavopereirine affected cell cycle progression. Figure 3A,B indicate that flavopereirine caused G2/M-phase cell cycle accumulation in HCT116 and DLD1 cells after 24 h treatment. An earlier study reports that P53-dependent P21 upregulation regulates G2-phase cell cycle progression by preventing the formation of the cell division control protein 2 /cyclin B1 complex [34]. Figure 3C discloses that flavopereirine upregulated the expression and Ser-15 phosphorylation of P53, indicating flavopereirine upregulated P53 activity to control cell cycle arrest. Moreover, flavopereirine enhanced P21 expression and diminished that of the G2/M regulatory cyclin B1. Furthermore, the Ser-10 phosphorylation of histone H3 involves in regulating G2/M progression [35]. Consistently, flavopereirine decreased pHH3 in HCT116 and DLD1 cells. These data (Figure 3) indicate that flavopereirine may upregulate P53 activity to prevent G2/M transition by increasing P21, decreasing intracellular cyclin B1, attenuating HH3 activity and resulting in arresting the G2/M-phase cell cycle.

### 2.4. Flavopereirine Lowers CRC Cell Viability via JAKs-STATs-c-Myc Signaling but Is Not Dependent on It

**Figure 4:**
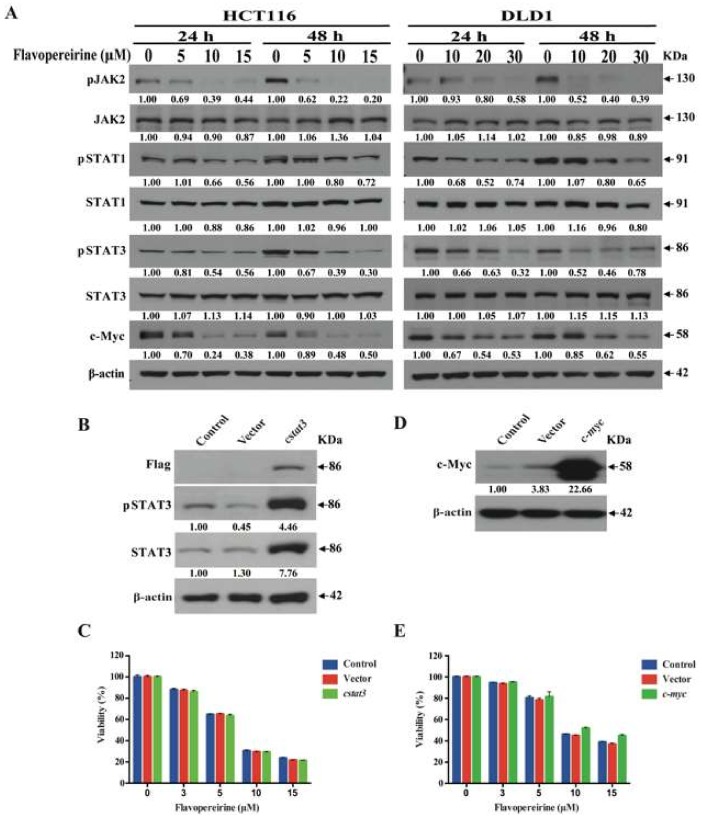
Flavopereirine non-dependently utilized STAT3/c-Myc signaling to reduce CRC cell viability. (**A**) Phosphorylation of JAK2, STAT1 and STAT3 and expression of downstream c-Myc protein were suppressed in a dose-dependent manner after treatment with flavopereirine for 24 h and 48 h. (**B**,**D**) STAT3 and c-Myc proteins were strongly upregulated by introducing *cstat3* and *c-myc* into CRC cells at 48 h. (**C**,**E**) The enforced expressions of STAT3 and c-Myc did not reverse flavopereirine-mediated viability reduction in CRC cells. Numbers under the plots indicate the fold change of protein level relative to their untreated control. Similar results were obtained for at least three independent experiments.

The JAKs/STATs pathway is involved in CRC progression [14]. For this reason, the effects of flavopereirine on JAKs-STATs-c-Myc signaling were examined. Figure 4A shows that the phosphorylation of JAK2, STAT1, and STAT3 and the expression of downstream c-Myc protein were suppressed in a dose-dependent manner after 24 h and 48 h flavopereirine treatment. Therefore, the JAK2-STAT1/STAT3-cMyc signal axis might participate in the flavopereirine-induced reduction of CRC cell viability. The importance of this signaling pathway was underscored by inducing the expression of constitutive active STAT3 and c-Myc in CRC cells. The stable transfection of constitutive active *stat3* (*cstat3*) increased STAT3 expression and phosphorylation (Figure 4B). However, constitutive active STAT3 expression did not reverse the reduction of CRC cell viability by flavopereirine treatment (Figure 4C). Moreover, the transient transfection of *c-myc* strongly upregulated c-Myc (Figure 4D). On the other hand, enforced c-Myc expression failed to reverse the reduction of CRC cell viability by flavopereirine treatment as well (Figure 4E). Figure 4 reveals that flavopereirine lowered CRC cell viability through the JAKs-STATs-c-Myc pathway, but was independent of STAT3-c-Myc signaling.

### 2.5. P53 Signaling Is Involved in Flavopereirine-Mediated Viability Reduction and Apoptosis Induction in CRC Cells

**Figure 5:**
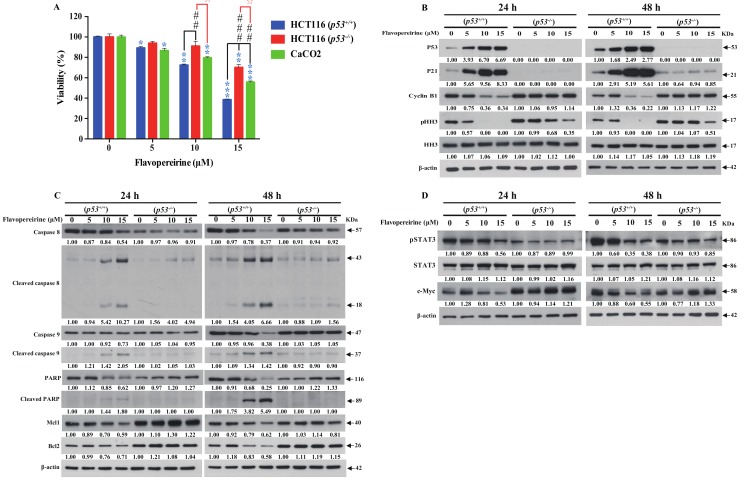
P53 signaling participated in flavopereirine-mediated viability reduction and apoptosis induction. HCT116 *p53^+/+^*, *p53^−/−^* and CaCO_2_ cells were treated with flavopereirine for 48 h. (**A**) Cell viability was determined by CCK8 assay. Each value was the mean ± SD of quadruplicate assays. * *p* < 0.05; ** *p* < 0.01, *** *p* < 0.001 compared to each untreated control. ^##^ *p* < 0.01 and ^###^ *p* < 0.001 compared to flavopereirine (10 or 15 µM) treated HCT116 (*p53^+/+^*) group. ^☆^*p* < 0.05 and ^☆☆^*p* < 0.01 compared to flavopereirine (10 or 15 µM) treated HCT116 (*p53^−/−^*) group. (**B**–**D**) Pro-apoptotic, anti-survival, G2/M-phase cell cycle, and STAT3 signaling were not changed in HCT116 (*p53^−/−^*) cells relative to wild type HCT116 (*p53^+/+^*) cells after flavopereirine treatment for 24 h and 48 h. Numbers under the plots indicate the fold change of protein level relative to their untreated control. Similar results were obtained in at least three independent experiments.

P53 expression and activity play important roles in restricting malignant cell growth by inducing cell cycle arrest and apoptosis [36]. Flavopereirine increased P53 expression and phosphorylation in CRC cells in a dose-dependent manner. Here, we verified the functional involvement of P53 in the suppression of CRC cell viability by flavopereirine treatment. To this end, we used wild type HCT116 (*p53*^+/+^), isogenic *p53* knockout HCT116 (*p53*^−/−^), and P53-expressing CaCO_2_ which generates a nonfunctional, undetectable truncated P53 protein due to a UAG nonsense mutation at codon 204 of *p53* gene [37]. Figure 5A shows that HCT116 (*p53*^−/−^) and CaCO_2_ cells were more resistant to flavopereirine than HCT116 (*p53*^+/+^) cells after 48 h treatment. Therefore, functional P53 expression is vital in flavopereirine-mediated CRC cell growth suppression.

Flavopereirine upregulated P21 and downregulated cyclin B1 in HCT116 (*p53*^+/+^) cells, but not in HCT116 (*p53*^−/−^) or CaCO_2_ cells (Figure 5B and Supplementary Figure S2A). Thus, p53-dependent p21 upregulation is crucial in flavopereirine-mediated G2/M-phase cell cycle arrest. Moreover, flavopereirine-activated apoptosis was induced in HCT116 (*p53*^+/+^) cells, but not in HCT116 (*p53*^−/−^) or CaCO_2_ cells (Figure 5C and Supplementary Figure S2B). Flavopereirine also suppressed STAT3 phosphorylation and c-Myc expression in HCT116 (*p53*^+/+^) cells, but not in HCT116 (*p53*^−/−^) or CaCO_2_ cells (Figure 5D and Supplementary Figure S2C). Figure 5 and Supplementary Figure S2 demonstrate that functional P53—not the JAKs-STATs-c-Myc signaling axis—regulates flavopereirine-mediated growth suppression. Interesting, HH3 phosphorylation was decreased, but not entirely repressed, in HCT116 (*p53*^−/−^) or CaCO_2_ cells (Figure 5B and Supplementary Figure S2A), implying an independent mechanism of flavopereirine-mediated HH3 phosphorylation during G2/M-phase cell cycle progression.

### 2.6. Flavopereirine Significantly Inhibits HCT116-Xenograft Tumor Growth In Vivo

**Figure 6:**
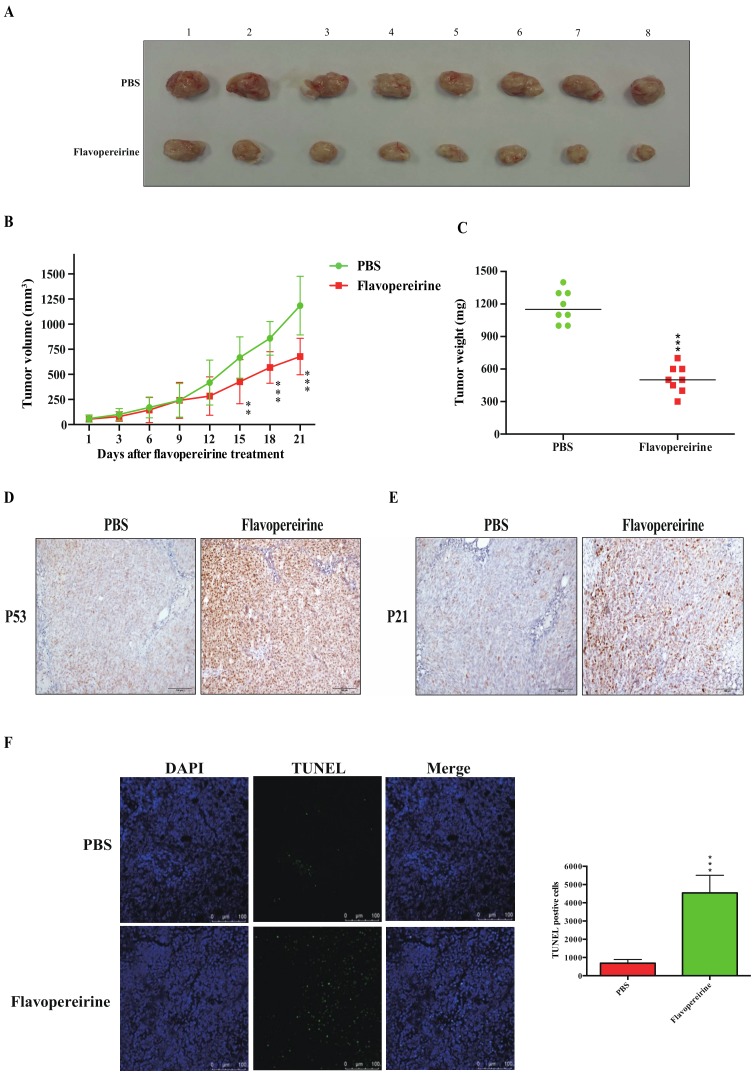
Flavopereirine significantly inhibited in vivo HCT116-xenograft growth. HCT116-bearing mice were treated either with PBS (*n* = 8) or flavopereirine (10 mg kg^−1^ d^−1^) (*n* = 8). (**A**) Representative images of excised tumors from each group. (**B**) Tumor volumes were measured to reflect tumor growth. ** *p* < 0.01, *** *p* < 0.001 compared with PBS. (**C**) Tumor weights were compared on the last day of treatment. *** *p* < 0.001 compared with PBS. (**D**,**E**) P53 and P21 expression levels in HCT116 tumors were evaluated by immunohistochemical staining. Scale bar: 100 µm. (**F**) Apoptosis in HCT116 tumors was measured by TUNEL staining. *** *p* < 0.001 compared with PBS. Scale bar: 100 µm.

Flavopereirine significantly affected CRC cell viability. For this reason, the therapeutic efficacy of flavopereirine on HCT116 xenograft tumor growth was studied in vivo. Figure 6A depicts HCT116 xenograft tumorsFigure 6B,C reveal that flavopereirine substantially suppressed HCT116 xenograft tumor growth according to tumor volume and weight after three weeks of flavopereirine treatment. Figure 6D,E disclose that P53 and P21 were markedly upregulated in HCT116 xenograft tumors treated with flavopereirine compared with those subjected to phosphate-buffered saline (PBS; control). We also examined TUNEL (terminal deoxynucleotidyl transferase dUTP nick end labeling)-stained cells inside the tumors. These are indicative of apoptosis. Figure 6F shows that relative to the PBS-treated controls, flavopereirine significantly enhanced apoptosis in HCT116 xenograft tumors. Furthermore, the effect of flavopereirine on normal colon tissue in vivo was also examined. By consulting with pathologist, Supplementary Figure S3 shows that the structure of colon wall was intact without significant morphological alternation and the infiltration of inflammatory cells within the colon wall was not found after flavopereirine treatment in mouse colon tissue in vivo, indicating the specific toxicity of flavopereirine on CRC cells rather than normal colon cells. The in vivo assay results presented in Figure 6 were consistent with those obtained and reported for the in vitro data, namely, flavopereirine-mediated CRC growth inhibition proceeds via P53-P21 signaling and apoptosis induction.

## 3. Discussion

CRC is an aggressive malignant tumor associated with high global mortality [38]. Surgery and chemotherapy are first-line treatments, but patients nonetheless present with recurrences and metastasis [39,40]. Therefore, low-toxicity, highly efficacious therapeutic agent are urgently needed for CRC. The present study demonstrated that flavopereirine markedly suppressed CRC cell growth in a dose-dependent manner via P53-, but not JAKs-STATs-c-Myc-dependent signaling. This mechanism arrested the G2/M-phase cell cycle and promoted apoptosis.

Our data also revealed that flavopereirine reduced CRC cell viability at the more highly malignant stages. This finding was consistent with that reported for a previous study in which the flavopereirine-based agent PB100 inhibited the proliferation of various malignant cells, but not normal cells [31]. The reason might be due to the flavopereirine preferentially impeding the initiation of DNA synthesis [30] during the growth of highly proliferative cancer cells rather than normal cells or slow-growing tumor cells.

The P53 and JAKs-STATs-c-Myc signals are significantly involved in CRC cell growth [41]. However, it is not understood why flavopereirine functions are mostly P53-dependent rather than STAT3- or c-Myc-dependent, as P53 is upstream of the latter two. An earlier work [30] reports that β-carboline alkaloids including flavopereirine selectively repress DNA synthesis in vitro by forming alkaloid-DNA complexes with cancer cells, but not with normal cells. This complex inhibits synthesis rather than chain elongation in cancer cell DNA [30]. P53 causes cell cycle arrest in response to DNA damage. It also interferes with DNA replication by directly interacting with the DNA replication machinery [42]. These results [30,42] imply that by hindering the initiation of DNA synthesis in CRC cells, flavopereirine may upregulate P53 activity, and trigger cell growth arrest, apoptosis induction, and the downregulation of survival signals including JAKs-STATs-c-Myc. This mechanism functionally restrains the growth of CRC and other cancer cells. Furthermore, STAT3 phosphorylation and c-Myc expression were diminished in HCT116 (*p53^+/+^*) but not in HCT116 (*p53^−/−^*) or CaCO_2_ cells after flavopereirine treatment, indicating P53 is upstream of JAKs-STATs-c-Myc signaling. P53 upregulation by the flavopereirine-DNA complex might be largely responsible for the suppression of CRC cell growth. However, the molecular mechanism by which the flavopereirine-cancer DNA complex promotes P53 expression remains to be elucidated.

Flavopereirine treatment strongly upregulated P53, but its mode of action is uncertain. A previous study indicates that the transcriptional regulation and post-translational modification of P53 occur simultaneously during DNA damage, nevertheless, the post-translational modification of P53, including protein phosphorylation and ubiquitination, is a faster and more sensitive means of P53 protein level regulation than transcriptional control [43]. *p53* mRNA expression is controlled by various transcriptional regulators including microRNAs, *p53* anti-sense RNA wrap, and epigenetic regulation [44]. Moreover, during DNA damage, post-transcriptional and post-translational modifications interfere with P53 protein expression and stability. The post-transcriptional regulation of P53 by various RNA-binding proteins is essential for the modulation of P53 activity. Earlier studies show that RNA-binding proteins promote [45] or repress [46] P53 translation by binding to the 5´ or 3´ untranslated regions of *p53* mRNA. Post-translational modifications including P53-MDM2 protein interaction [47] and P53 phosphorylation [48] regulate P53 stabilization and degradation. MDM2, a P53-specific E3 ubiquitin ligase, restricts P53-mediated growth suppression in unstressed cells [49]. Radiation-induced Ser-15 phosphorylation in P53 [50] prevents P53 proteasomal degradation from MDM2 and P53 interaction [51]. The aforementioned results suggest that transcriptional, post-transcriptional, and post-translational regulation are possibly involved in controlling flavopereirine-mediated P53 expression, but the detailed mechanisms involved remain to be determined.

Flavopereirine significantly reduced cell viability and enhanced apoptosis in both wild type and mutant CRC cells harboring functional P53 expression. On the other hand, it had no such influences on nonfunctional P53-expressing CRC cells. Conventional chemotherapies against advanced CRC raise P53 activity, arrest the cell cycle, inhibit DNA replication, and cause cell death [52]. *P53* mutation is observed in ~35% to 40% of all CRC cases. The efficacies of chemotherapy [53] and radiotherapy [54] are significantly reduced by mutations in the DNA-binding domain, which results in P53 protein conformational change and decreases the transcriptional activity of P53, as these diminish the interaction between P53 and DNA. Therefore, restoration of normal P53 function and expression can sensitize CRC to these therapies. Missense P53 mutations are associated with conformational change and cause the accumulation of misfolded protein in the nucleus. Thus, P53 reactivation is an effective cancer therapy strategy. An earlier study reveals that treatment with the mutant P53 activator chetomin causes P53 to refold into a wild type-like conformation and induces apoptosis in various cancer cells [27]. Furthermore, the reactivation of P53 and the induction of tumor cell apoptosis (RITA) binds P53, blocks its interaction with MDM2, enhances and stabilizes the capacity of P53 to induce apoptosis, suppresses cancer cell growth [28,55], and may sensitize CRC cells to chemotherapy [26]. Flavopereirine could selectively treat CRC bearing functional—but not nonfunctional—p53 expression. However, it is still unclear as to how flavopereirine induces apoptosis in mutant P53-expressing CRC cells by causing a conformational change in mutant P53, enhancement of net P53 function, and upregulation of the mutant P53 protein level.

*Kras* mutations occur in ~35% to 40% of all CRC cases [56]. *Kras* mutations in CRC are predictive markers for anti-epidermal growth factor receptor therapy resistance [57,58] and clinical responsive markers for platin-based treatment. Therefore, *Kras* status is crucial in CRC therapy. Our data indicated that flavopereirine reduced the viability of *Kras* wild type (HT-29) and *Kras* mutant (SW480, SW620, DLD1, and HCT116) cells. Moreover, a previous study discloses that the both *Kras* wild type and mutant non-small lung cancer cells are sensitive to the β-carboline alkaloid krukovine which mediates growth suppression [59]. This finding is consistent with our results and suggests that β-carboline alkaloids, such as flavopereirine, exhibit great potential to treat CRC bearing wild type and mutant *Kras* [59].

## 4. Materials and Methods

### 4.1. Cell Culture

The CRC cell lines SW1116, SW480, SW620, DLD1, HT29, and CaCO_2_ were purchased from the Bioresource Collection and Research Center (BCRC; Hsinchu, Taiwan). SW1116, SW480 and SW620 cells were cultured in Leibovitz L-15 Medium (Life Technologies, Grand Island, NY, USA). HT29 and DLD1 cells were cultured in high-glucose Dulbecco’s modified Eagle’s medium (Invitrogen, Carlsbad, CA, USA). CaCO_2_ cells were cultured in Eagle’s minimum essential medium (Invitrogen). Wild type HCT116 (*p53^+/+^*) cells and isogenic *p53* null cells (HCT116 (*p53^−/−^*)) were purchased from Horizon Discovery Group (Cambridge, UK) [60] and cultured in RPMI 1640 medium (Invitrogen). All media were supplemented with 10% fetal bovine serum (FBS; Invitrogen) and antibiotics (100 U mL^-l^ penicillin and 100 mg mL^-l^ streptomycin) according to the manufacturer’s instructions.

### 4.2. Flavopereirine

Flavopereirine (CAS Number 70128-63-1) with molecular formula (C_17_H_15_N_2_ClO_4_) and molecular weight (346.76 g/moL) was obtained from ChromaDex (Irvine, CA, USA). After purchasing, flavopereirine was dissolved with dimethyl sulfoxide and stored at −20 °C. Furthermore, the dissolved flavopereirine was diluted in complete cell culture medium for experiments.

### 4.3. Viability Assay

CRC cell lines (5 × 10^3^ well^−1^) were seeded in a 96-well plate and treated with various concentrations of flavopereirine in culture medium for 24 h and 48 h. Then the cell proliferation reagent WST-1 (Roche Applied Science, Penzberg, Germany) was added to determine cell viability [61].

### 4.4. Apoptosis Assay

Fluorescein isothiocyanate (FITC)-labeled annexin-V and propidium iodide (PI) (BD Biosciences Inc., San Jose, CA, USA) were applied as a double stain to evaluate flavopereirine-induced apoptosis. CRC cells were treated with various concentrations of flavopereirine in culture medium for 48 h, washed and stained with annexin V-FITC and PI, and analyzed by flow cytometry. The percentage of annexin-V-positive (early apoptosis) and annexin-V + PI-positive (late apoptosis) cells was calculated in CellQuest (BD Biosciences Inc., San Jose, CA, USA).

### 4.5. Western Blotting Analysis

The effects of flavopereirine on protein expression and phosphorylation, apoptosis, cell cycle, and signal transduction were verified by western blotting using specific antibodies. CRC cells were seeded in culture plates, treated with various concentrations of flavopereirine for 24 h and 48 h, and lysed. Cellular protein was then extracted and quantified. Equal concentrations of cellular protein (20~30 µg) were subjected to SDS-PAGE electrophoresis for western blotting. Antibodies against P21 (1:2000), cyclin B1 (1:1000), Bcl-2 (1:1000), cdc2 (1:1000), Ser-10 phosphorylated histone H3 (pHH3) (1:1000), and histone H3 (1:3000) were purchased from Santa Cruz Biotechnology (Dallas, TX, USA) and used to reveal cell cycle progression. Antibodies against caspase-3 (1:2000), -8 (1:2000), -9 (1:2000), PARP (1:2,000), cleaved caspase-3 (1:1000), -8 (1:1000), and -9 (1:1000), cleaved PARP (1:1000), Mcl-1 (1:1000), Bid (1:1000), and Bim (1:1000) were purchased from Cell Signaling Technology (Danvers, MA, USA) and used to determine cellular apoptosis. The following were applied to disclose flavopereirine-affected signal transduction pathways: antibodies against P53 (1:2000) (Santa Cruz Biotechnology, Dallas, TX, USA), Ser-15 phosphorylated P53 (1:1000) (pP53; Cell Signaling Technology, Danvers, MA, USA), Tyr-1007/1008 phosphorylated JAK2 (1:2000) (pJak2; Cell Signaling Technology, Danvers, MA, USA), JAK2 (1:4000) (Cell Signaling Technology, Danvers, MA, USA), Tyr-701 phosphorylated STAT1 (1:2000) (pSTAT1; Cell Signaling Technology, Danvers, MA, USA), STAT1 (1:4000) (Epitomics, Burlingame, CA, USA), Tyr-705 phosphorylated STAT3 (1:2000) (pSTAT3; Cell Signaling Technology, Danvers, MA, USA), STAT3 (1:1000) (Cell Signaling Technology, Danvers, MA, USA), and c-Myc (1:1000) (Cell Signaling Technology, Danvers, MA, USA). Anti-β-actin antibody (1:5000) (Santa Cruz Biotechnology, Dallas, TX, USA) served as a loading control.

### 4.6. Cell Cycle Analysis

PI (BD Biosciences Inc., San Jose, CA, USA) staining was used to verify the effects of flavopereirine on CRC cell cycle progression. CRC cells were starved in serum-free medium for 24 h to synchronize their cell cycles, treated with various concentrations of flavopereirine for 24 h, collected and fixed for PI staining, and analyzed by flow cytometry. The percentage of cells in G1, S, and G2/M was determined in ModFit (BD Biosciences Inc., San Jose, CA, USA).

### 4.7. Enforced Expression of Constitutive Active STAT3 (cSTAT3) and c-Myc in CRC Cells

The lentivirus-based constitutive active *stat3* (*cstat3*)-expressing plasmid *pcstat3* (EF.STAT3C.Ubc.GFP, 24983) was purchased from addgene (Watertown, MA, USA). To generate viral particles, *pcstat3* was transfected into HEK293T cells. The cSTAT3-expressing lentiviral supernatant was collected [62] and used to infect the CRC cells. The green fluorescence protein (GFP) -positive CRC cells were separated and used in subsequent experiments.

The c-Myc expressing plasmid *pc-myc* (pcDNA3-myc, 16011) purchased from addgene (Watertown, MA, USA) was applied to induce c-Myc overexpression in CRC cells. The *pc-myc* plasmid was transiently transfected into HCT116 [63]. Both cSTAT3- and c-Myc-overexpressing HCT116 cells were treated with flavopereirine for 48 h. The cell proliferation reagent WST-1 (Roche Applied Sciences, Penzberg, Germany) was added to determine cell viability.

### 4.8. In Vivo Tumor Growth Assay

To evaluate the effects of flavopereirine on CRC growth in vivo, male mice with severe combined immunodeficiency (eight weeks; BioLASCO, Taipei, Taiwan) were used with the approval of the Institutional Animal Care and Use Committee of Chang Gung Memorial Hospital (Chiayi, Taiwan). Briefly, HCT116 cells (10^6^) were subcutaneously injected into the mice. Mice bearing tumors (~100 mm^3^ volume) were assigned to PBS or flavopereirine (10 mg kg^−1^ mouse^−1^) treatment groups. The materials were injected intraperitoneally once daily. Tumor volume was measured every 3 d and was not allowed to grow over 2000 mm^3^. After 21 d, the tumors were harvested, photographed, fixed, and frozen for future analysis.

### 4.9. Immunohistochemical and Immunofluorescent TUNEL Staining Assays of HCT116-Xenograft Tumor

Immunohistochemical staining assays were performed as described in a previous study [64]. Briefly, HCT116 tumor sections (diameter 10 µm) were stained with rabbit anti-P53 and anti-P21 antibodies (Santa Cruz Biotechnology, Dallas, TX, USA). An immunofluorescent TUNEL-staining kit (No. 11767291910; Sigma-Aldrich Corp., St Louis, MO, USA) was used to detect apoptosis in the CRC-xenograft tumor according to the manufacturer’s (Roche Applied Science, Penzberg, Germany) instructions. DAPI (4′,6-diamidino-2-phenylindole) nuclear staining served as a counterstaining control. TUNEL-positive cells were quantified at five randomly selected fields (×400 magnification) per tissue slide in quintuplicate assays (ImageJ; NIH, Bethesda, MD, USA). Furthermore, the morphology of colon tissue sections was examined by pathologist.

### 4.10. Statistical Analyses

GraphPad Prism v. 5 (GraphPad Software, La Jolla, CA, USA) was used to perform the data analyses. Data were presented as means ± standard deviation (SD). Statistically significant differences between one group were assessed by unpaired Student’s *t*-tests. Differences between more than two groups were compared by two-way analysis of variance and following Bonferroni post hoc test, with values of *p* less than 0.05 considered statistically significant.

## 5. Conclusions

The molecular mechanism by which flavopereirine reduced cell viability, arrested the cell cycle, and induced apoptosis in CRC was proposed in supplementary Figure S4. The present study revealed that flavopereirine activated P53-P21, rather than JAKs-STSTs-cMyc signaling, to suppress CRC cell growth through the extrinsic and intrinsic apoptotic pathways and G2/M-phase cell cycle arrest.

## Supplementary Materials

The following are available online at https://www.mdpi.com/2072-6694/11/7/1034/s1, Figure S1: Oxaliplatin reduced the viability of various CRC cell lines, Figure S2: P53 signaling was involved in flavopereirine-mediated viability reduction and apoptosis induction in truncated and nonfunctional P53-expressing CRC cells, Figure S3: Flavopereirine treatment did not significantly change the integrity of normal colon tissue in mouse in vivo, Figure S4: Proposed mechanisms of flavopereirine in cell cycle distribution and apoptosis for affecting CRC cell growth regulation.
