# Supplementary material for: Flavopereirine Suppresses the Growth of Colorectal Cancer Cells through P53 Signaling Dependence
Source: Cancers (Basel). 2019 Jul 22;11(7):1034. doi: 10.3390/cancers11071034 (PMC6678721; doi:10.3390/cancers11071034)
Supplement: Supplementary file 1 [file cancers-11-01034-s001.pdf]

Supplementary Materials: Flavopereirine Suppresses the Growth of Colorectal Cancer Cells through P53 Signaling Dependence

Jhy-Ming Li, Yun-Ching Huang, Yi-Hung Kuo, Chih-Chung Cheng, Feng-Che Kuan, Shun-Fu Chang, Ying-Ray Lee, Chih-Chien Chin and Chung-Sheng Shi

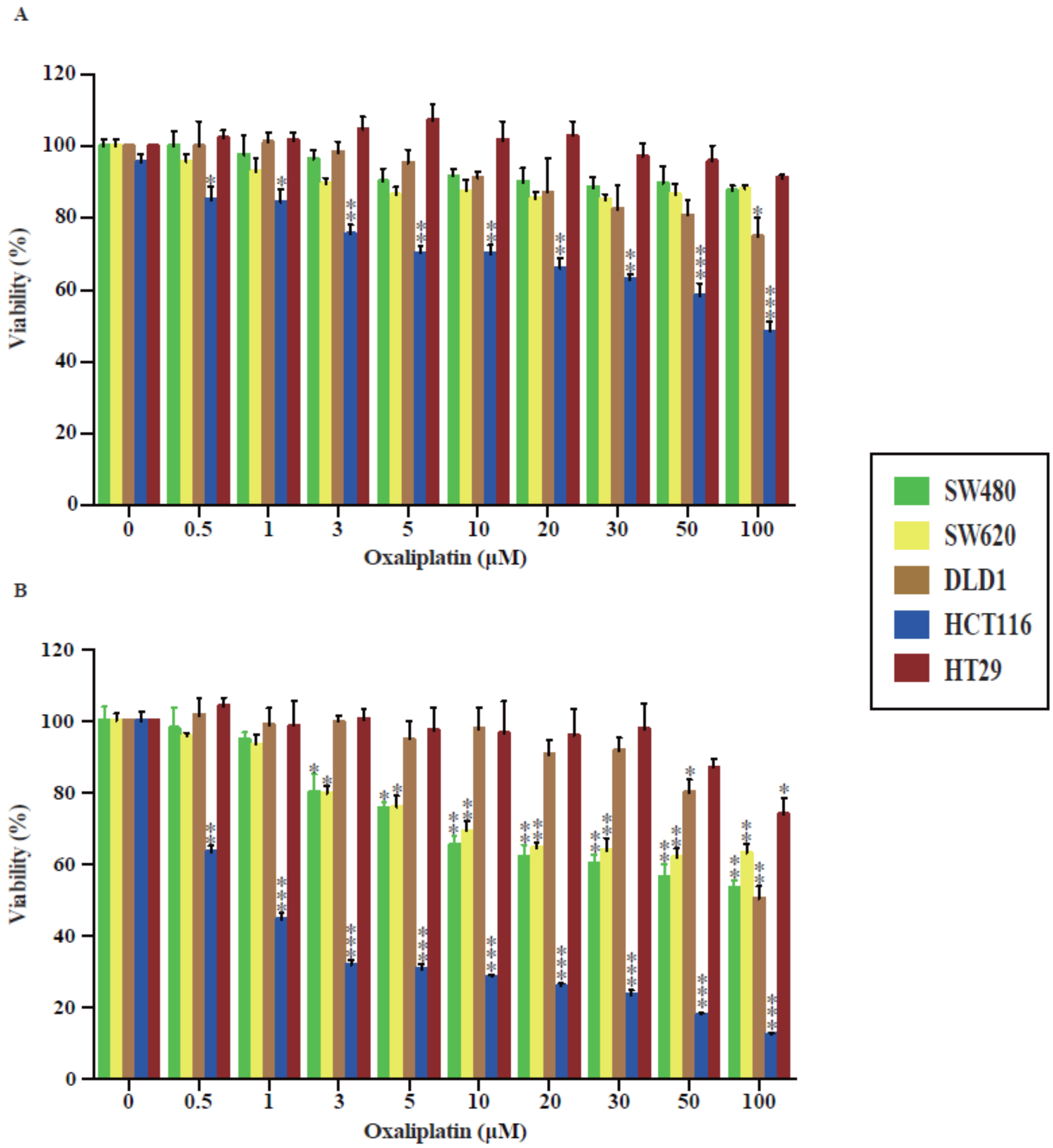

Figure S1. Oxaliplatin reduced the viability of various CRC cell lines. CRC cells were treated with various concentrations of oxaliplatin for 24 h and 48 h. CRC cell viability was then assayed by CCK8. Each value was the mean ± SD of quadruplicate assays. *$P < 0.05$; **$P < 0.01$, ***$P < 0.001$ compared with the control.

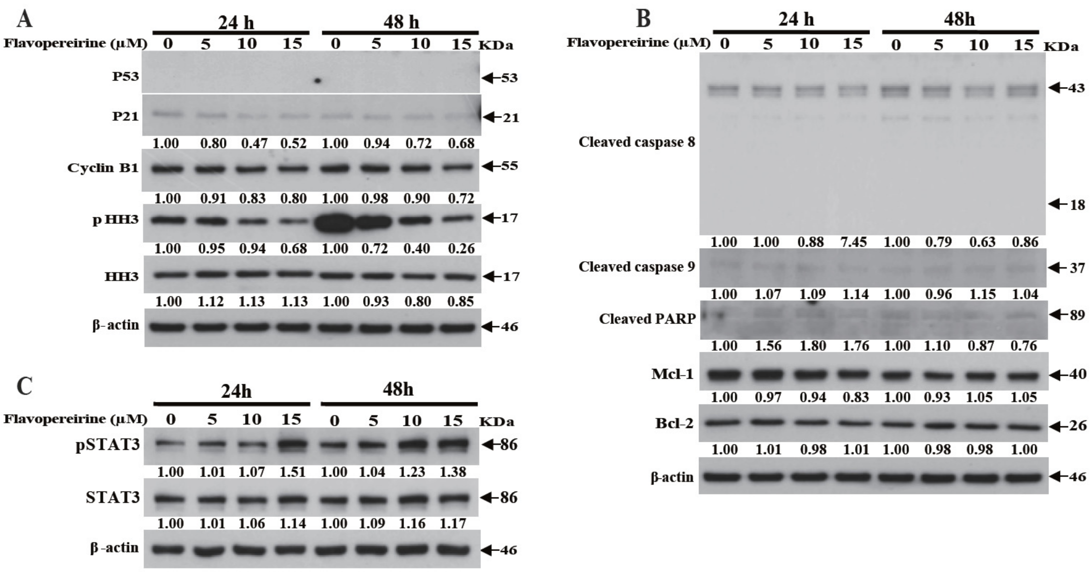

Figure S2. P53 signaling was involved in flavopereirine-mediated viability reduction and apoptosis induction in truncated and nonfunctional P53-expressing CRC cells. CaCO2 cells were treated with flavopereirine for 24 h and 48 h. A, B, and C. Pro-apoptotic, anti-survival, G2/M-phase cell cycle proteins, and STAT3 signaling were not changed in CaCO2 cells after flavopereirine treatment for 24 h and 48 h.

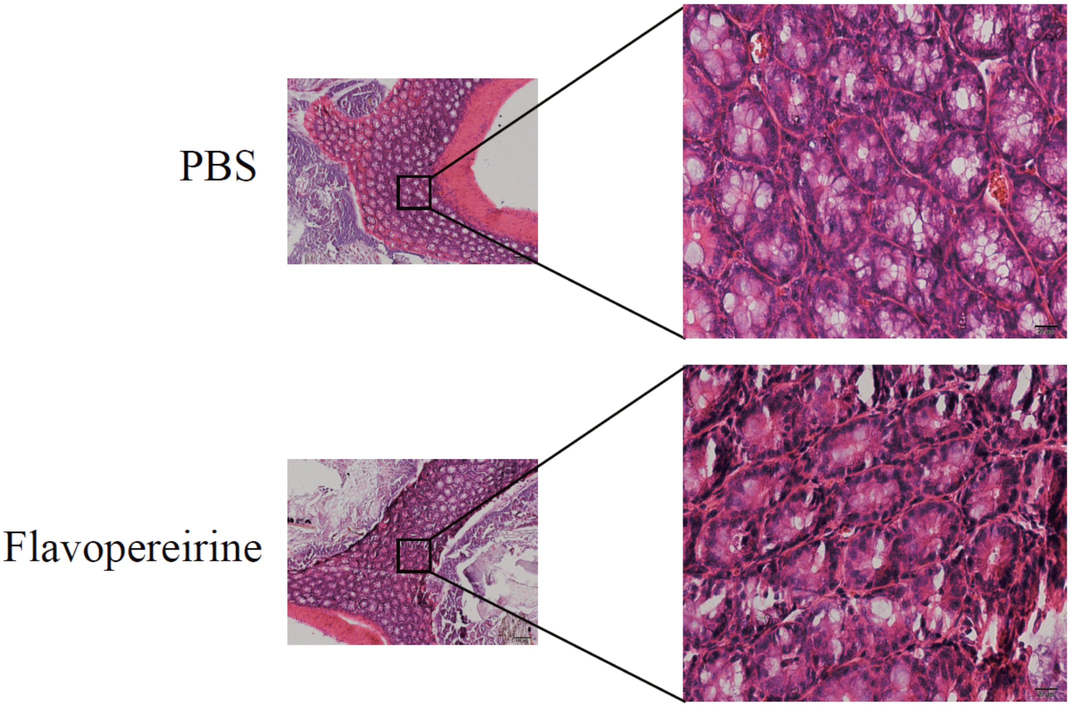

Figure S3. Flavopereirine treatment did not significantly change the integrity of normal colon tissue in mouse in vivo. Mouse was intraperitoneally injected with flavopereirine or PBS for 21 days. After treatment, the histology of mouse colon stained with haematoxylin/eosin was examined.

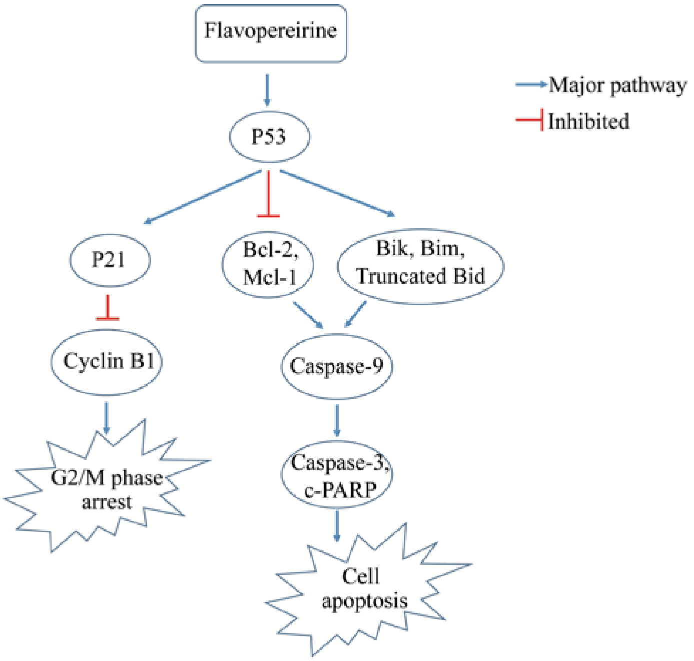

Figure S4. Proposed mechanisms of flavopereirine in cell cycle distribution and apoptosis for affecting CRC cell growth regulation. Flavopereirine upregulated P53 and P21 and downregulated cyclin B1, which prevented cyclin B1/Cdk1 cascade formation and G2/M-phase cell cycle arrest. P53-dependent signaling played critical roles in extrinsic and intrinsic flavopereirine-induced CRC cell apoptosis. Both P53-mediated G2/M-phase cell cycle arrest and apoptosis suppressed CRC growth.
